# Assessment of postoperative uterine isthmus thickness on MRI after surgical resection of retrocervical deep infiltrating endometriosis

**DOI:** 10.1007/s00261-025-04975-z

**Published:** 2025-07-02

**Authors:** Oriane Bernigaud, Gil Dubernard, Emmanuelle Maissiat, Dorothée Taconet, Audrey Haquin, Benoit De La Fourniere, Marion Cortet, Charles-André Philip

**Affiliations:** Hôpital de la Croix-Rousse, Lyon, France

**Keywords:** Endometriosis surgery, Torus uterinum, Uterine rupture, USL, Pregnancy outcomes

## Abstract

**Study objective:**

The aim of this study was to assess the impact of endometriosis surgery in the retrocervical area and/or USL on the thickness of the posterior uterine isthmus, and subsequently the potential risk of posterior uterine rupture during labor.

**Design:**

Retrospective observational study.

**Setting:**

Improving the management of patients with deep infiltrating endometriosis, keeping in mind potential obstetric consequences.

**Patients:**

All endometriosis patients treated surgically at the Croix-Rousse University Hospital during the study period for the resection of a lesion on one or both USL and/or in the retrocervical region were included if preoperative and postoperative MRI images were available in their medical record.

**Interventions:**

Evaluating the variation of posterior uterine isthmus thickness on MRI images after retrocervical surgery for deep infiltrating endometriosis. Searching for predictive markers of posterior uterine isthmus thinning.

**Measurements and mean results:**

Forty-two patients were included between 2016 and 2020. We observed a significant decrease in the sagittal thickness of the median posterior uterine isthmus after surgery (-2.25 mm, 95% CI -3.18 to -1.32, *p*<.01). A significant association was also found between the thinning of the median posterior uterine isthmus on the sagittal section and preoperative urinary symptoms (*p* =.04), a history of digestive resection with ileostomy (*p* =.02) and the presence of post-voiding residue ≥ 100 cc postoperatively (*p*<.01). We observed 6 pregnancies in 4 patients (9.5%). Four pregnancies were carried to term with cesarean delivery.

**Conclusion:**

The resection of retrocervical endometriosis is associated with postoperative thinning of the posterior uterine isthmus, which seems positively associated with the complexity of the rest of the surgery.

## Abstract (fr)

The laparoscopic resection of deep endometriosis lesions from the retrocervical area and/or USL leads to a significant 2.25 mm reduction of posterior uterine isthmus thickness on sagittal MRI.

## Graphical abstract

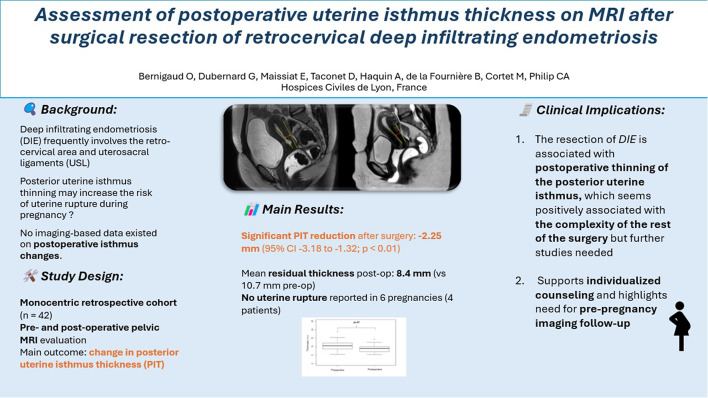

## Introduction

The retrocervical region and/or the uterosacral ligaments (USL) are the most frequent lesion locations in posterior deep infiltrating endometriosis [1]. Clinical manifestations include dysmenorrhea and dyspareunia and are typically resistant to hormone therapy, thus justifying a surgical approach [2]. Consequently, a significant number of women with retrocervical and/or USL lesions are treated with surgery while wishing for a pregnancy. Current data is insufficient regarding the obstetrical impact of pelvic endometriosis surgery in these women [3]. Uterine rupture is among the worst adverse event following uterine surgery. In the postoperative setting of deep infiltrating endometriosis, several cases of posterior uterine rupture were described, occurring before or during labor, though the frequency of the event after this type of surgery was not established [4–11]. There is no published data on the impact of posterior uterine surgery for endometriosis on the solidity of the scar tissue. The primary objective for this study was to assess if the excision of retrocervical endometriosis lesions was associated with posterior uterine isthmus thickness (PIT), leading in turn to a weakening of the uterus. A retrospective study was conducted to examine the MRI evolution of the posterior uterine isthmus and cervix after a surgical treatment of retrocervical and/or USL lesions, to appraise the risk of uterine weakening associated with these surgeries.

## Methods

In this monocentric retrospective study, all patients underwent a surgical resection of endometriosis lesions in the retrocervical region and/or on uterosacral ligaments (left, right or both) between 1st January 2016 and 31st December 2020 in the Gynecology Department of the Croix-Rousse University Hospital (Hospices Civils de Lyon and Lyon 1 University, France). Out of all endometriosis patients surgically managed during the study period, we recruited those with retrocervical lesions and/or lesions on one of the USL that had been resected. The uterosacral ligament lesions included in our study were all proximal, located at the uterine insertion of the uterosacral ligaments. Of those patients, we included those for whom preoperative MRI images and images from at least one postoperative pelvic MRI were available. Patients were excluded if they were aged under 18, if the data in their postoperative report was incomplete regarding the type of resection performed, if they had received a “conservative” surgical treatment (destruction, coagulation, vaporization, etc.) or if no MRI images were available. Other exclusion criteria were a history of hysterectomy, prior myomectomy or myomectomy performed within the same surgical procedure as the deep infiltrating endometriosis resection.

The following data was collected retrospectively from patient records: biometric data, previous pregnancies and births, history of surgical treatment for endometriosis, preoperative symptoms (if available, an assessment of pain symptoms was appreciated using a simple 0–10 visual analogue scale) and medical management of endometriosis in the 3 months leading up to the surgery. If several preoperative MRI images were available, measurements were taken on the set closest to the date of the surgery. Similarly, if the patient had had several postoperative MRIs, images from the first MRI to be performed after the surgery were used. MRI images were examined in T2W sequences and using sagittal section. Data was collected on the characteristics of posterior pelvic nodules, of the posterior uterine isthmus and of the cervix. Reports from any MRI performed in an external radiology center were included in the patient’s digital medical record, allowing for centralized and reproductible readings of the images using the software Centricity™ Universal Viewer, version 6.0, GE Healthcare. The surgical approach to the posterior uterine resection was documented, as well as postoperative complications (categorized as per the Clavien Dindo classification). Post-surgery follow-up included monitoring for the occurrence of pregnancy; in such an event, data was collected regarding the outcome, due date, mode of delivery and any uterine rupture.

**Fig. 1:**
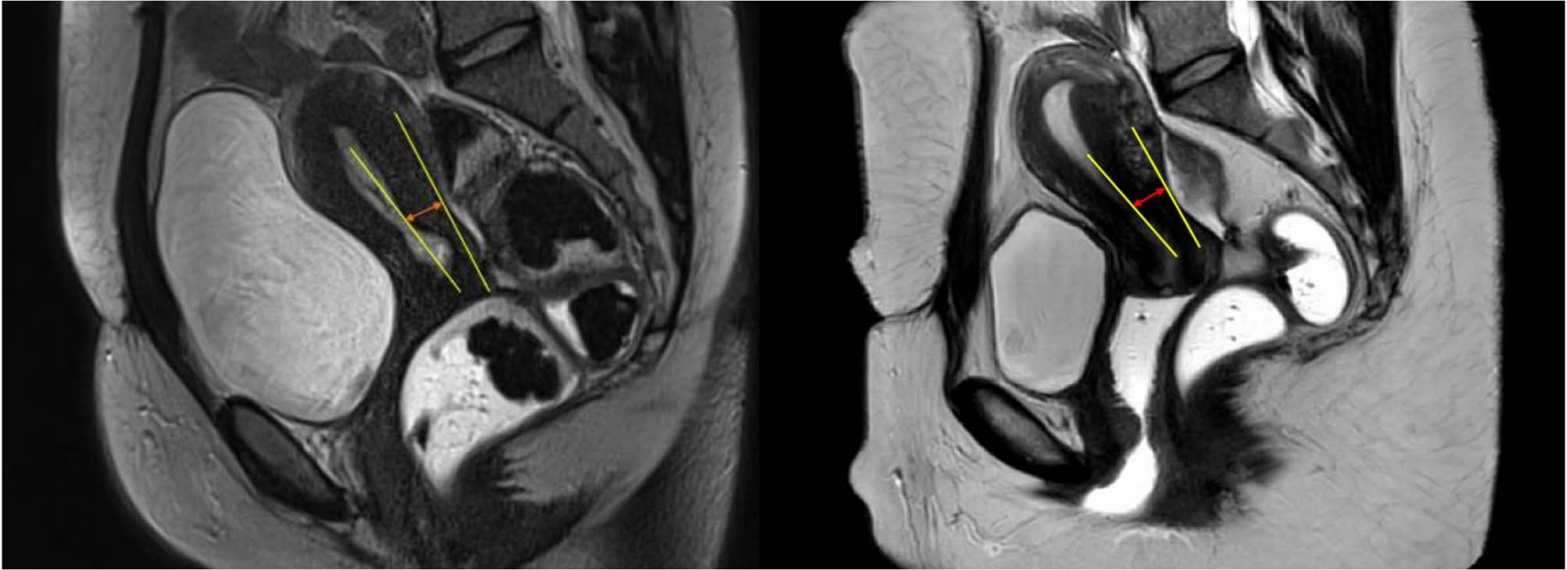
Examples of posterior isthmus thickness measurement on sagittal T2-weighted pelvic MRI. Yellow solid line: posterior uterine wall axis (endometrial and peritoneal edge) / Red arrow: posterior uterine isthmus thickness (millimeter)

Our primary endpoint was the comparison of the minimum thickness of the median posterior uterine isthmus on MRI sagittal sections before and after surgical resection of retrocervical and/or uterosacral ligament lesions. To achieve this endpoint, 2 radiologists (AH and DT) analyzed both preoperative and postoperative MRI images side by side blinded to the time of the procedure. The measurement of the isthmus thickness was conducted by identifying the myometrial layer and drawing a line connecting two tangent axes: one at the endometrial edge and the other at the peritoneal edge of the myometrium (as depicted in Fig. 1). Subsequently, the thickness measurement was taken between the outer serosal layer externally and the endometrium-myometrium junction internally. Lastly, the volume of the uterus cervix was compared on the MRI before and after surgery. A senior radiologist with an expertise in endometriosis offered a validation reading of the pelvic MRI images.

In view of identifying predictive markers for a weakening of the posterior isthmus (secondary objective), we searched for a correlation between the characteristics of the posterior isthmus (thickness, median volume) and the characteristics of the lesion, the type of surgery performed, the approach used, any complications, and the patient’s profile. Postoperative relapse was clinically defined as the recurrence of endometriosis-related symptoms, particularly pelvic pain, after surgery, excluding cases attributed to musculoskeletal disorders or central sensitization syndromes.

The collected data was centralized on a password-protected Excel (Microsoft for Windows) spreadsheet. A unique code was attributed to each patient to anonymize the data. A To define the descriptive data, we used the mean, standard deviation, number of subjects and percentage. The statistical analysis was performed using the “R” software (R Core Team (2020), R Foundation for Statistical Computing, Vienna, Austria). To compare the continuous preoperative and postoperative variables, a Student test for paired series was used. Factors associated with isthmus thickness reduction were researched using a univariate analysis with a simple linear regression.

All patients included in the study received an information letter explaining the research objectives and the nature of the collected data, at which point they had the right to withdraw from the study. The scientific and ethical committee for the Hospices Civils de Lyon (HCL) validated the study on 26/05/2021 (reference number 21_466).

The treatment of personal data within the study complies with the MR-004 referencing methodology (Méthodologie de Référence n°4) from the Commission Nationale de l’Informatique et des Libertés (CNIL) with which the HCL have a compliance agreement, as well as the EU’s General Data Protection Regulation (GDPR).

## Results

### Sample characteristics

**Fig. 2:**
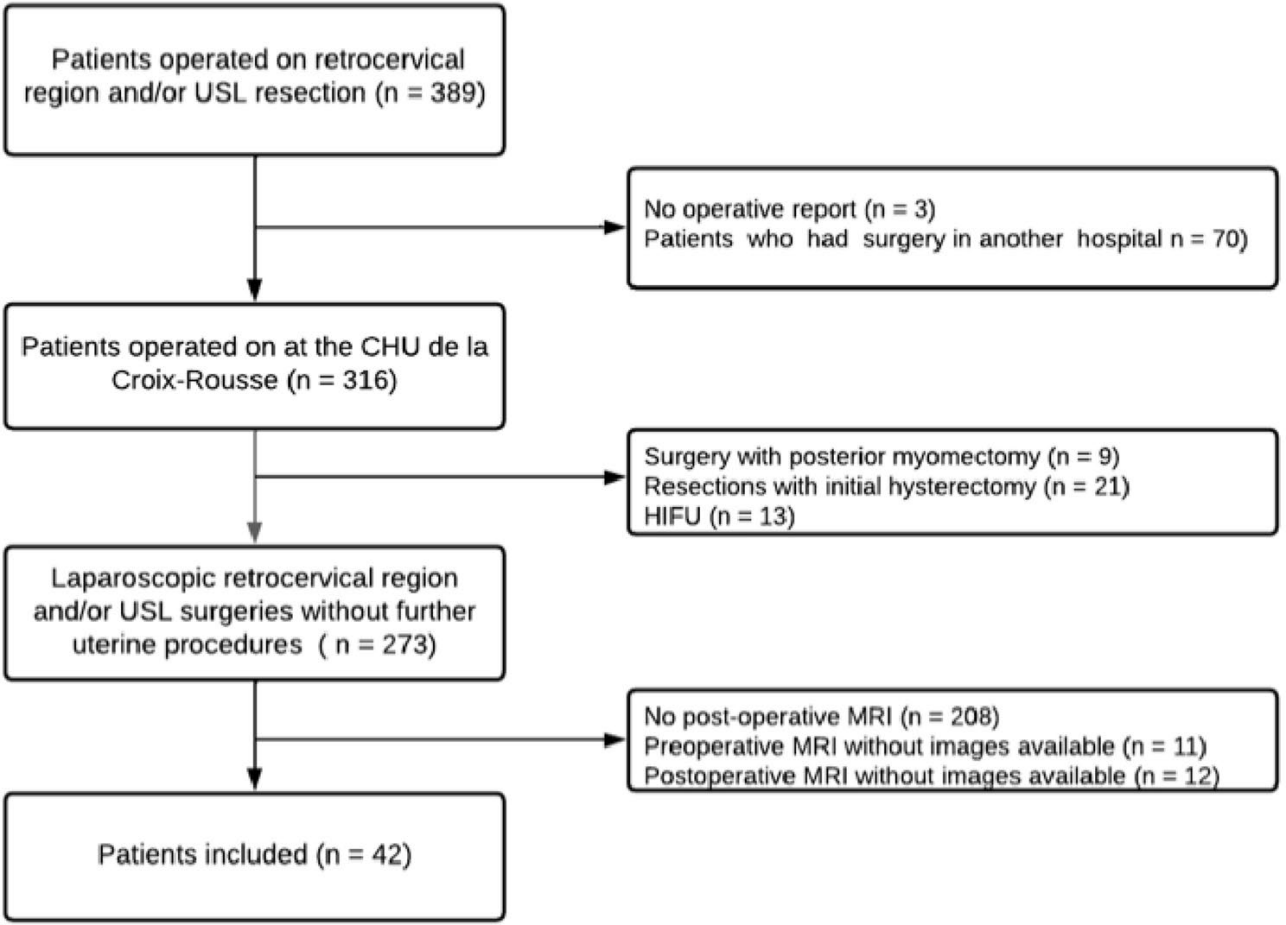
Flow chart

The study included forty-two patients who underwent a surgical resection of retrocervical and/or uterosacral ligament lesions at the Croix-Rousse university hospital between January 2016 and August 2020. For all patients, a preoperative MRI was performed in various radiology centers between January 2015 and February 2020. Twenty-seven (64%) of the preoperative MRIs were performed in the radiology department of the Croix-Rousse university hospital. All patients also had a postoperative MRI between March 2018 and July 2021, 3 to 50 months after surgery (mean: 14 months, median: 11 months). Thirty-one (74%) of those MRIs were performed in the radiology department of the Croix-Rousse university hospital. The study flow chart can be seen in Fig. 2. None of the eligible patients refused to take part. Patient characteristics are summarized in Table 1. In this cohort, the mean maximum diameter of the posterior endometriosis nodule was 24.4 mm (SD = 10.4 mm) and its mean volume 3.7mm^3^ (SD = 3.7mm^3^). Respective median values were 23.0 mm and 2.0mm^3^. Patients received surgery between 1 and 24 months after the preoperative MRI, with a mean waiting time of 7.3 months (SD = 5.8 months). On sagittal sections, the assessment of the median posterior isthmus thickness (PIT) after surgery could be performed in 41 patients (97.6%).

**Table 1:** Patients characteristics (*N* = 42)

Characteristics	Mean ± SD (median) [min-max]
Age (years)	35.4 ± 5.8 (37) [24–47]
BMI (kg/m^2^)	22.9 ± 4.23 (23) [16–35]
Number of pregnancies prior to surgery	0.93 ± 1.31 (0) [0–5]
Number of births (≥ 22 WA) prior to surgery	0.71 ± 1.11 (0) [0–4]
Preoperative surgical history	n (%)
Endometriosis surgical history	17 (40.5)
Superficial peritoneal endometriosis	10 (23.8)
Endometrioma	10 (23.8)
Deep infiltrating endometriosis (digestive, urologic, vaginal)	0 (0)
Retrocervical/uterosacral ligament lesions	2 (4.8)
History of anterior isthmus scarring (cesarean section)	4 (9.5)
Preoperative functional signs	n (%)
Digestive functional signs	29 (69.1)
Urological functional signs	13 (31)
Chronic pelvic pain	31 (73.8)
Dysmenorrhea	39 (92.9)
Deep dyspareunia	34 (80.9)
Hormone therapy in the 3 months prior to surgery	n (%)
Estrogen + progestogen	10 (23.8)
Low-dose or high-dose progestins	15 (35.7)
GnRH analogs	8 (19.1)

A significant reduction of the size of the endometriosis nodule was observed after surgery, with no residual lesion on the MRI and/or no postoperative relapse in 19 patients. This result was observed both in the volume of the nodule (-3.19mm^3^; 95% CI = [-4.28; -2.09]; *p*<.01) and in its maximum diameter (-18.6 mm; 95% CI = [-23.09; -14.10]; *p*<.01).

### Sagittal section median PIT

**Fig. 3:**
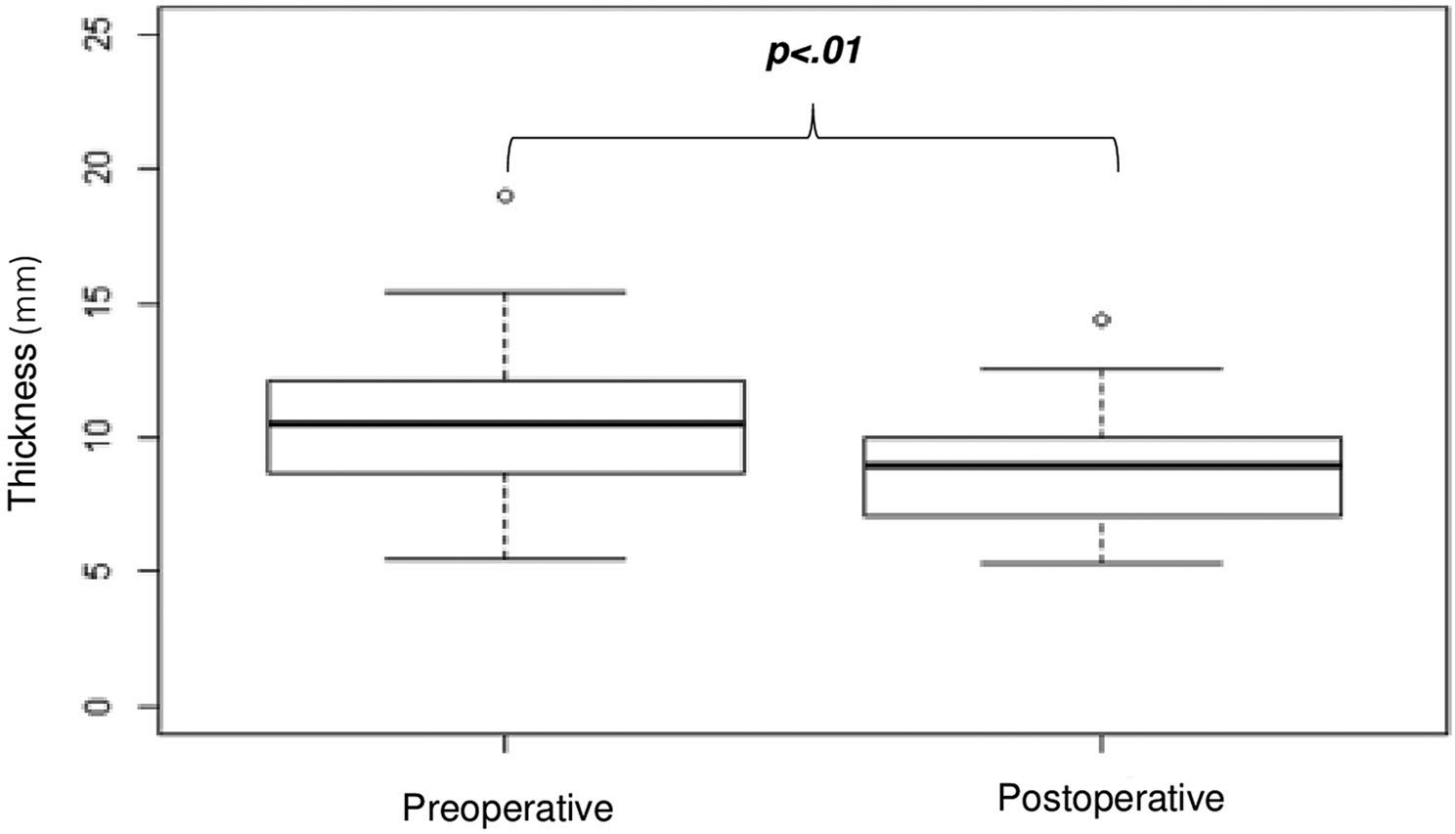
Comparison of median sagittal thickness before and after surgery on pelvic MRI

On a sagittal section, the surgical resection of a retrocervical and/or uterosacral ligament endometriosis nodule was associated with a significant reduction of median PIT (-2.25 mm; 95% CI = [-3.18; -1.32]; *p* <.01) (Fig. 3).

### Uterine volume cervix comparison

**Fig. 4:**
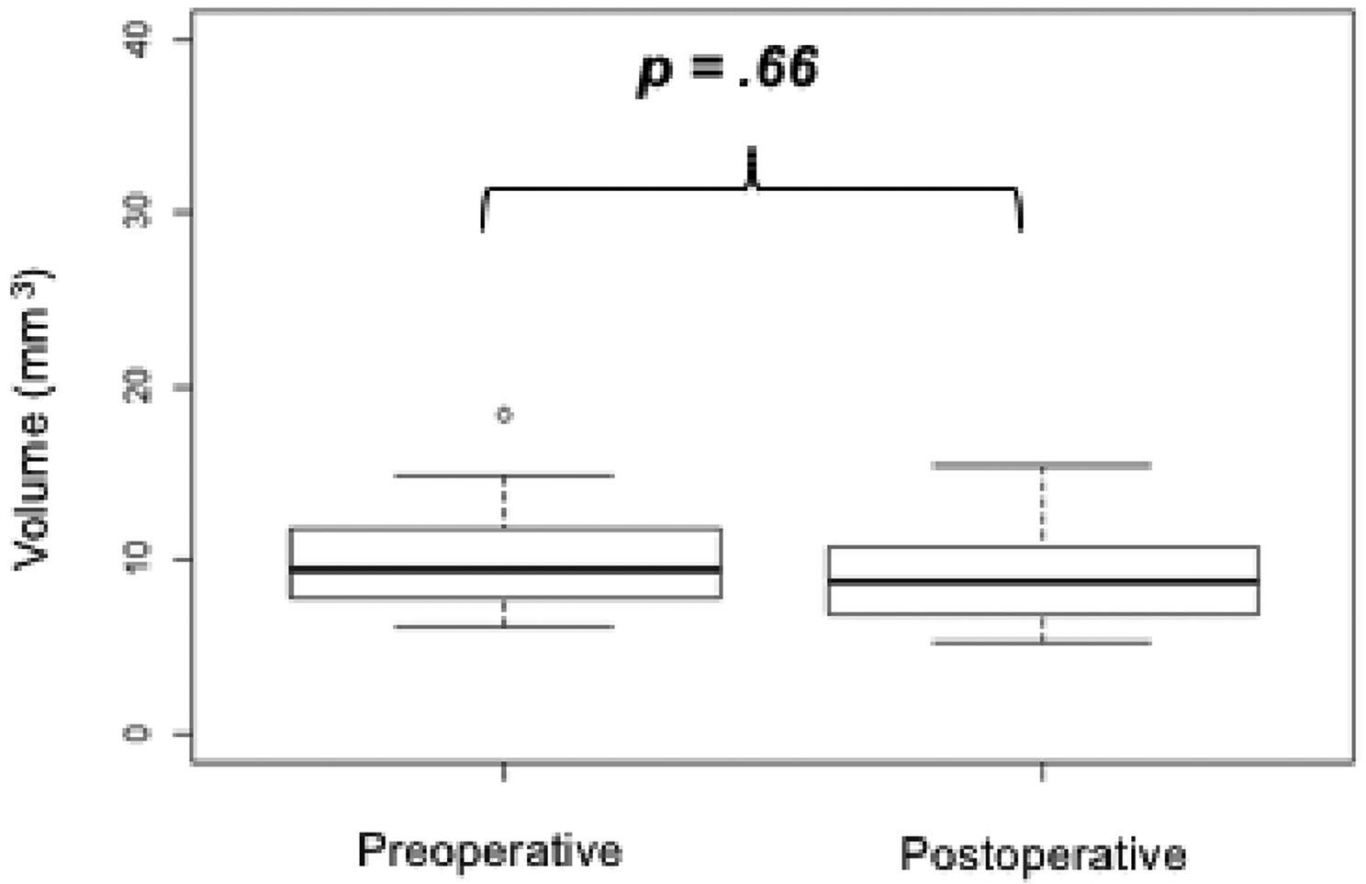
Comparison of cervical volume before and after surgery on pelvic MRI

No statistically significant variation of the volume of the cervix was evidenced after retrocervical and/or uterosacral ligament resection (+ 0.90mm^3^; 95% CI = [-3.23;5.05]; *p* =.66). Table 2; Fig. 4 summarize the compared measurements of PIT and cervical volume before and after surgery.

**Table 2:** Comparison of posterior isthmus and cervix MRI characteristics MRI before and after surgery

	*n*	Preop MRI (mean [95% CI])	Postop MRI (mean [95% CI])	Difference [95% CI]	*p*-value
Median PIT on sagittal section (mm)	41	11.0 [9.8; 12.3]	8. 4[7.4; 9.3]	-2.25 [-3.18; -1.32]	<0.01
Cervical volume (mm^3^)	39	9.1 [8.0; 10.2]	10.0 [5.7; 14.2]	+ 0.90 [-3.23; 5.05]	0.66

CI: confidence interval, preop: before surgery, postop: after surgery

### Factors associated with PIT

In the univariate analysis, the reduction of the posterior isthmus thickness on the sagittal section was significantly associated with retrocervical resection compared to uterosacral ligament resection (slope *a* = -2.00; *p* =.05). In addition, median PIT reduction on the sagittal section was positively correlated with preoperative urinary symptoms (*a* = -2.03; *p* =.04), performing a digestive resection with ileostomy (*a* = -3.29; *p* =.02), the presence of post-micturition residue ≥100 cc after the surgery (*a* = -16.45; *p*<.01) and the occurrence of hemorrhagic complications (*a* = -16.45; *p* <.01). No cases of bowel perforation or ureteral injury occurred within our cohort. As a result, these complications were not included in the analysis, given their absence. Median PIT reduction on the sagittal section was also significantly associated to preoperative cervical volume (*a* = -0.45; *p* <.01) and lesion size (volume and maximum diameter, respectively *a* = -0.34; *p* <.01 and *a* = -0.15; *p* <.01). After cervical volume adjustment (*p* <.01), we could still observe a positive association between the volume of the lesion and the extent of PIT reduction on the sagittal section (*a* = -0,2 mm), though no longer significant (*p* =.08).

The patients’ history of endometriosis surgery (superficial, endometrioma, retrocervical/uterosacral ligament resection), age, weight, and number of previous pregnancies and births were not statistically associated with median PIT variation on the sagittal section nor left or right paramedian PIT variation.

Table 3 shows median PIT variation on the sagittal section ian based on the type of resection performed.

**Table 3:** PIT variation based on resection type (simple linear regression)

	Retrocervical resection *n* = 31	Right uterosacral ligament resection *n* = 29	Left uterosacral ligament resection *n* = 35
Median PIT variation on sagittal section *n* = 41	-2.00 (SD = 0.99) *p* =.05	-0.94 (SD = 0.99) *p =*.35	-0.72 (SD = 1.23) *p =*.56

In the univariate analysis, whether the uterosacral ligament resection was unilateral or bilateral or whether a concomitant retrocervical/uterosacral ligament resection had been performed was not significantly associated with median PIT reduction on the sagittal section (respectively *p* =.30 and *p* =.78). Concomitant retrocervical/uterosacral ligament resection was not significantly associated with the maximum diameter of the lesion (*p* =.08) but it was significantly associated with a larger reduction of the volume of the nodule after surgery (*p* =.02).

### Secondary pregnancies

We reported 6 pregnancies in 4 of the 42 patients included in the study (9.5%). Due to the retrospective nature of the study, we were unable to determine the number of patients desiring pregnancy during the study follow-up period. However, we note that 5 out of the 6 pregnancies (83.33%) were achieved through IVF. Pregnancies started between 8 days and 34 months after surgery. One patient suffered a miscarriage; her subsequent pregnancy culminated at 24 weeks of amenorrhea (WA) with the vaginal delivery of a child who subsequently died in neonatal care due to extreme prematurity. The other 4 pregnancies were carried to term with cesarean section deliveries. An indication was present in 2 patients: a prophylactic section was scheduled for a child presenting in a breach position and an emergency section was performed due to abnormal fetal cardiac rhythm during labor induction with prostaglandin, in the context of severe intrauterine growth restriction at 37 WA. No uterine rupture was reported.

## Discussion

In this retrospective study comprising 42 patients who underwent surgical treatment for deep infiltrating endometriosis involving retrocervical and/or uterosacral ligament lesions, we observed a significant reduction in both thickness and volume of the median posterior isthmus on MRI scans post-surgery. Our case series is the first in the existing literature to document changes in posterior isthmus thickness before and after surgery, providing insight into the risk of uterine weakening. Therefore, there is limited opportunity for direct comparison between our findings and those of other published case series. However, our results are not surprising as it has long been established that uterine isthmus surgery, such as cesarean section, is associated with postoperative isthmus thickness reduction [12–14]. The literature review from Tanos et al. examining 52 studies on uterine rupture evidence that a reduction in the thickness of the myometerium in relation to the cesarean section scar on the uterus outside of a pregnancy is a likely predictive factor for uterine rupture [15].

The clinical implications of our primary finding, particularly regarding subsequent pregnancies, are currently undefined. Van der Voet et al. reported a decrease in the mean anterior uterine isthmus thickness of at least 2 mm following cesarean section, as measured by ultrasound [16]. These results are in line with our observations, which are surprising when considering the low rate of isthmus opening. Our estimations show that median PIT on the sagittal section was on average 11 mm before surgery and 8.4 mm after surgery (-24%). The available literature does not report a potential physiological threshold for uterine wall thickness. In this retrospective study, no instances of uterine rupture were reported, which makes it difficult to establish a direct correlation between the observed postoperative isthmus thickness and a potential threshold indicative of uterine rupture risk. While the reduction in isthmus thickness warrants consideration, it is obvious that the average residual thickness (8.4 mm) in our series appears reassuring compared to the 3 mm threshold by Tanos et al. as a risk of uterine ruptures following cesarean Sect. [15]. However, such comparisons remain subject to caution, as it is difficult and probably inaccurate to extrapolate the impact of uterine thickness reduction in the anterior wall to the one in the posterior wall. Indeed, it is unclear whether the anterior and posterior cervical walls serve the same function or behave similarly during pregnancy. Indeed, the association between a threshold thickness of the anterior isthmus and clinical impact remains undefined to date even after cesarean Sect. [17].

Our study had several biases, primarily due to its retrospective nature, which constrained the number of factors investigated and the reliability of data collection. The inclusion of only 42 patients also limited the scope of our analyses, particularly in exploring associations between posterior isthmus thickness (PIT) reduction and various factors. The inability to measure the posterior uterine isthmus in one patient was due to technical limitations during MRI imaging, specifically suboptimal anatomical visualization caused by patient positioning or movement. We chose to exclude this patient rather than risk overestimating the measurement due to potential artifacts. The restricted patient sample size was attributed to the low availability of postoperative imaging, as MRI scans are typically recommended in cases of suspected complications or recurrence. Another of the study’s limitations is that there were no blinded measurements taken on the MRI in relation to the type of surgery performed or the MRI report. In addition, not all measurements could be taken on the total sample number. The limitations in interpreting the images lie in the lack of a systematic orthogonal section through the isthmus and in the fact, we did not have 3D reconstruction tools at our disposal. We also acknowledge that 22% of our patients did not receive any hormonal treatment prior to surgery, and this information is not available in post-operative MRI data. The potential variation of the myometrial junction with the hormonal cycle could therefore introduce a specific bias. Moreover, it should be acknowledged that one limitation of our study is that the interval between surgery and postoperative MRI was not included as a covariate in the statistical analysis, which may have influenced the measurement of posterior isthmus thickness. Although not statistically significant, the slight increase in cervical volume observed postoperatively may reflect either a limitation in measurement precision or subtle postoperative tissue remodeling at the periphery of the cervix. In patients with endometriosis, who are frequently women of reproductive age, any uterine surgery should consider its potential obstetric consequences. Several case reports have described secondary uterine ruptures following the surgical management of deep infiltrating endometriosis [4–11]. There is also the question of a weakening of the uterus due to the lesion itself, which is evidenced by several cases of a spontaneous rupture of the uterine artery that have been described in relation to an endometriosis nodule as soon as the second trimester of pregnancy in patients with no history of surgery. Another example pointing to the likelihood of an intrinsically weakened uterus is the case of a spontaneous uterine rupture in an adenomyosis patient with no history of surgery during a twin pregnancy [18]. Based on our results and the examples provided in the literature, it may be prudent for surgeons to lean towards a more conservative resection strategy, minimizing tissue removal where possible, or to carefully reconsider the necessity of extensive surgery, particularly in the retrocervical region. There is a need for prospective studies to investigate the correlation between preconception posterior isthmus thickness observed on imaging and pregnancy outcomes, to formulate more precise recommendations. While assessing data using different ultrasound techniques would have been relevant and cost-effective, such data were not available for our retrospective study. Nevertheless, in a prospective study, evaluating the posterior isthmus via sonography before and during pregnancy could be insightful for understanding its behavior in relation to the elongation of the posterior wall during pregnancy.

## Conclusion

This unicentric retrospective study over a 4-year period evidences a significant reduction of the sagittal thickness of the posterior uterine isthmus (2.25 mm) after the surgical resection of retrocervical and/or uterosacral ligament endometriosis lesions. The real obstetrical impact of this thinning, namely regarding the risk of uterine rupture, remains to be determined. It should however be considered in the follow-up of any endometriosis patient who becomes pregnant.

## Data Availability

No datasets were generated or analysed during the current study.
